# 1195. "The Economic Impact of Antimicrobial Use for Surgical Prophylaxis: A Prospective Study

**DOI:** 10.1093/ofid/ofad500.1035

**Published:** 2023-11-27

**Authors:** James Gnanamani, Ratheesh Rajakumar, Suresh Kumar Dorairajan

**Affiliations:** Jaya College of Paramedical sciences, chennai, Tamil Nadu, India; Jaya College of Paramedical Sciences, chennai, Tamil Nadu, India; Apollo hospital, Chennai, Tamil Nadu, India

## Abstract

**Background:**

Antibiotic prophylaxis is commonly used to prevent surgical site infections in patients. However, excessive use of antibiotics can lead to adverse effects such as the development of antibiotic resistance and increased healthcare costs.Unless there is a known infection, prophylactic antibiotics should be discontinued within 24 hours.This study aimed to evaluate the cost implications of antibiotic prophylaxis in patients who received prophylaxis for a duration longer than recommended by the World Health Organisation (WHO).

**Methods:**

This prospective observational study was conducted in a tertiary care hospital in India for a period of two months. Data on patient demographics and Surgical antimicrobial usage were collected to surgical patient cases. Follow-up was performed to determine whether the antibiotics has been stopped after 24 hours. Financial implications, such as increase in drug costs were documented when the surgical prophylaxis was continuing after 24 hours. The necessity of antimicrobial usage and its cost effectiveness were analysed using basic statistical method.

**Results:**

This study included 174 patients of surgical antimicrobial therapy. Out of these, 58 patients (33%) had their surgical antimicrobial therapy stopped after 24 hours, while 116 patients (66%) continued to receive surgical antimicrobial therapy beyond the recommended duration. The data was then analysed, with a focus on the cost of the antimicrobial medication (as shown in Table 1).

**Figure:**
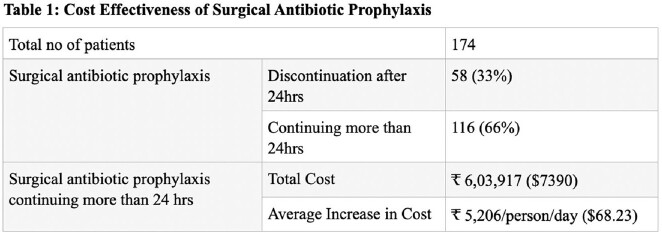

**Conclusion:**

The study revealed that when surgical antibiotic prophylaxis was administered for more than 24 hours, the average expenditure per person per day increased by ₹5206 ($68.83). Additionally, patients who received antibiotic prophylaxis beyond the recommended duration incurred significantly higher costs than those who received prophylaxis for the recommended duration, with the cost of antibiotics accounting for the majority of the excess expenditure. These findings emphasise the potential cost savings associated with reducing the duration of antibiotic prophylaxis to the recommended duration, without compromising patient outcomes. Further research is necessary to evaluate the cost-effectiveness of implementing this practice.

**Disclosures:**

**All Authors**: No reported disclosures

